# The Role of Self-Blaming Moral Emotions in Major Depression and Their Impact on Social-Economical Decision Making

**DOI:** 10.3389/fpsyg.2013.00310

**Published:** 2013-06-03

**Authors:** Erdem Pulcu, Roland Zahn, Rebecca Elliott

**Affiliations:** ^1^Neuroscience and Psychiatry Unit, Manchester Academic Health Sciences Centre, School of Medicine, The University of Manchester, Manchester, UK; ^2^Neuroscience and Aphasia Research Unit, Manchester Academic Health Sciences Centre, School of Psychological Sciences, The University of Manchester, Manchester, UK

**Keywords:** guilt, shame, major depressive disorder, neuroeconomics, social-economical decision making, neuroimaging

## Abstract

People with major depressive disorder (MDD) are more prone to experiencing moral emotions related to self-blame, such as guilt and shame. DSM-IV-TR recognizes excessive or inappropriate guilt as one of the core symptoms of current MDD, whereas excessive shame is not part of the criteria for MDD. However, previous studies specifically assessing shame suggested its involvement in MDD. In the first part of this review, we will consider literature discussing the role of self-blaming moral emotions in MDD. These self-blaming moral emotions have been purported to influence people when they make social and financial decisions in cognitive studies, particularly those using neuroeconomical paradigms. Such paradigms aim to predict social behavior in activities of daily living, by using important resource tangibles (especially money) in laboratory conditions. Previous literature suggests that guilt promotes altruistic behavior via acting out reparative tendencies, whereas shame reduces altruism by means of increasing social and interpersonal distance. In the second part of this review, we will discuss the potential influence of self-blaming moral emotions on overt behavior in MDD, reviewing clinical and experimental studies in social and financial decision-making, in which guilt, and shame were manipulated. This is not a well-established area in the depression literature, however in this opinion paper we will argue that studies of moral emotions and their impact on behavioral decision-making are of potential importance in the clinical field, by linking specific symptoms of a disorder to a behavioral outcome which may lead to stratification of clinical diagnoses in the future.

*But they whose guilt within their bosoms lie, Imagine every eye beholds their blame, For Lucrece thought he blush’d to see her shame*.

*The Rape of Lucrece, William Shakespeare*

## Introduction

Moral emotions (e.g., guilt, shame, indignation, prosocial forms of pride, gratitude) are discussed as of a critical evolutionary importance (Gintis et al., 2008). It has been suggested that humans acquired the capacity to feel these emotions over the course of evolution to motivate behavior that is directed toward other people’s or societal needs, thereby promoting social cooperation (Zahn et al., 2012). The relationship between moral emotions and social behavior has been of interest to philosophers since the Classical periods. More recently the relationship has been considered by clinicians and social psychologists, and moral emotion has become a “hot topic” in neuroscience, with the emergence of “social neuroscience” as a distinct discipline (Ochsner and Lieberman, 2001).

Healthy functioning of a moral emotion system forms the basis of balancing selfish needs with those of other people. Its dysfunction can lead to certain types of psychopathology. For example, lack of moral emotions such as sympathy and guilt has been mentioned among the key personality traits of psychopathic individuals (Hare, 1985; Mahmut et al., 2008; Haji, 2010). By contrast, the experience of self-blaming moral emotions, such as guilt, shame, and self-contempt/disgust can be exaggerated in mood disorders (Zahn et al., 2012). In the case of major depression, guilt is often exaggerated, and experienced out of context (Prosen et al., 1983), and is recognized as one of the core symptoms: “feelings of worthlessness or excessive or inappropriate guilt nearly every day (not merely self-reproach or guilt about being sick)” (American Psychiatric Association, 2000).

In the first part of this opinion paper (See Self-blaming Moral Emotions in Major Depressive Disorder and Hypotheses Regarding the Impact of Self-Blaming Emotions on Social-Economical Decision Making), we will discuss the role of self-blaming moral emotions (guilt and shame) in major depressive disorder (MDD). There is emerging evidence for the role of self-contempt/disgust in MDD (Green et al., 2013), but this is not further reviewed because the relative paucity of evidence. We will focus on reviewing evidence from behavioral and neuroimaging studies in people with clinical diagnoses of major depression, and will only briefly mention some of the studies in healthy populations with and without symptoms. It is beyond the scope of this paper to provide an overview of studies of guilt and shame in populations that were not assessed using diagnostic interviews. A recent meta-analysis has addressed this issue and provided evidence for the relationship between guilt, shame, and depression in patient populations as well as individuals reporting depressive symptoms (Kim et al., 2011). In the second part (See A Brief Introduction to Neuroeconomical Paradigms onward), we will discuss the impact of these self-blaming moral emotions on decision-making. Considering neuroeconomical studies in clinical, subclinical and healthy populations with mood induction, we will aim to highlight the potential implications of their findings for social-economical decision making in major depression. For the purposes of clarity, the present review will use “major depression” only when referring to studies conducted in people with clinical diagnoses of depression.

## General Clinical Aspects of Major Depressive Disorder

Major depression is a complex disorder with impairments in cognitive, emotional, and neurobiological domains. Various etiological models suggest that a combination of developmental, psychosocial, neurobiological, and genetic factors cause MDD (Kendler et al., 2006; Sjoholm et al., 2009). The key symptoms of major depression are persistent low mood, severe reduction in energy, and interest, intense feelings of worthlessness, hopelessness and; in about half of the patients, also excessive guilt. These symptoms are observed cross-culturally (Sartorius et al., 1980). Overall these features make depression a leading cause of disability in the western world (Eaton et al., 2008). Lifetime prevalence of depressive disorders is estimated at 16% in the UK (Sahakian et al., 2010) rising to 17.1% in the urban UK population (Ayuso-Mateos et al., 2001). The annual cost of mental health disorders reach as high as £77 billion in the UK when secondary costs arising from broader impacts such as reduced work efficiency are considered (Beddington et al., 2008). These figures, which are representative of the prevalence and social cost of depression throughout the developed world, justify the need for continuous, interdisciplinary research on depression, focusing on factors that constitute vulnerability and as well as factors that promote resilience to MDD.

## Self-Blaming Moral Emotions in Major Depressive Disorder

There are several self-conscious moral emotions (e.g., guilt, shame, and prosocial forms of pride; (Tangney, 2002), and it has been argued that certain of these are selectively enhanced in MDD (Zahn et al., 2012). Guilt and shame can be defined as “self-blaming moral emotions,” as distinct from other moral emotions such as indignation, related to blaming others (other-blaming) or self-praising moral emotions such as pride. Vulnerability to MDD has been associated with elevated levels of self-blaming emotions compared with other-blaming emotions (Green et al., 2013) as predicted by attributional models of MDD (Abramson et al., 1978).

### The role of guilt in major depressive disorder

Excessive or out of context (inappropriate) guilt has been recognized as one of the distinctive clinical symptoms of MDD, especially of the melancholic subtype (American Psychiatric Association, 2000). In a developmental psychological study in healthy children, it was shown that excessive or inappropriate guilt becomes less normative with age, as children make less predictive errors in explaining cause and effect relationships (Tilghman-Osborne et al., 2012). These authors suggest that excessive or inappropriate guilt becomes exponentially depressotypic with age. A recent meta-analysis showed that levels of guilt on the Hamilton Depression scale were higher in younger adults with current major depression compared with older adults (Hegeman et al., 2012). It is unclear, however, how representative the included studies were of the respective younger and older populations. Another study showed that parental guilt induction is more significant than the influence of parental depressive symptoms on the way children internalized their parent’s problems, which may be an additional vulnerability feature for adult MDD in these high-risk populations (Rakow et al., 2011). The symptom profiles of female patients with recurrent MDD, suggest that worthlessness and excessive guilt are the most discriminating factors for patients with and without history of suicide attempts (Bi et al., 2012). Other previous studies showed that patients with current MDD have significantly elevated levels of guilt measured on scales which conceptualized guilt in different ways: “delusional” and “affective” (Berrios et al., 1992), “state” and “trait” (Ghatavi et al., 2002) and “survivor” and “omnipotent responsibility” guilt (O’Connor et al., 2002).

A subtype of guilt that has been consistently reported in clinical populations as an exaggerated moral emotion (Blacher, 2000; O’Connor et al., 2002) is “survivor guilt,” associated with perceiving oneself as being better off than others (O’Connor et al., 2000). Historically, this concept gained clinical recognition during the Korean War, where it was noticed that when grenade explosions claimed multiple lives, the survivors subsequently became vulnerable to severe depression (Blacher, 2000). Similar observations of “post-survival” guilt were also recorded among the survivors of World War II (O’Connor et al., 2000) and patients undergoing treatment in an intensive care unit following successful organ transplantation (Blacher, 2000). In the post-operative cases mentioned by Blacher (2000), survivor guilt originates from limited availability of compatible donors helping a limited number of patients to recover, whereas leaving others on the waiting lists. Other forms of combat-related guilt significantly predict MDD diagnosis, especially when it arises from inactions in the face of observations of abusive violence, probably due to violations of one’s own moral conduct (Marx et al., 2010). Survivor guilt scores were elevated in people with major depression treated as inpatients and predicted the severity of depression (O’Connor et al., 2002).

Survivor guilt scores might have further predictive value in identifying populations who are vulnerable to future major depressive episodes. One recent study showed that scores for survivor and omnipotent responsibility guilt (defined as taking responsibility for events which may be out of one’s control and feeling guilty about their consequences) were significantly higher in a population of people with MDD fully remitted from symptoms compared with healthy subjects (Green et al., 2012). However, the same sample did not show elevated shame scores. These findings suggest that elevated guilt levels may be a trait vulnerability feature, or alternatively a scar following previous episodes of MDD. Survivor guilt may be an important construct that links research in the area of major depression to post-traumatic stress disorder (PTSD), disorders with a high degree of co-occurrence. Taken together, the studies in this section suggest that MDD is characterized by significantly higher levels of guilt, even when the symptoms are fully remitted.

### The role of shame in major depressive disorder

Whilst considering the role of shame in MDD, it is important to note that despite its proposed significance, exaggerated shame is not considered a diagnostic symptom of MDD (by contrast with guilt). Tangney and colleagues have investigated the role of shame in predicting depressive symptoms in healthy people using the Test of Self-Conscious and Affect (TOSCA) scale, which assesses indirect manifestations of affect on behavior. Their research group conceptualized shame with attempts to deny, hide or escape the shame-eliciting situation; as a result leading to interpersonal separation and increased social distance (Tangney et al., 2007). They showed that shame-proneness accounts for a substantial quantity of variance in depressive symptoms to an extent that this pattern cannot be reduced to an attributional style (Tangney et al., 1992b). However, they found these results in populations with no clinical diagnosis of depression (Tangney et al., 1996, 1998). Mild depressive symptoms in healthy populations could be due to a variety of reasons other than major depression suggesting a lack of specificity. Furthermore, although social withdrawal is an important clinical aspect of MDD, it may be more fundamental to other psychiatric disorders such as social phobia or borderline personality disorder (Rusch et al., 2007).

While shame-proneness may not be specific to depression, there is increasing evidence that it is clinically relevant. In clinical populations, it was shown that shame responses in the face of everyday social dilemmas were elevated in current major depression and that shame-proneness as a trait increased the risk of recurrence (Andrews, 1995; Thompson and Berenbaum, 2006). A recent study found a significant relationship between shame responses and severity of depression, and specifically suicidal ideation, more strongly than elevated levels of guilt (Bryan et al., 2013). Another study showed that patients with recurrent major depressive episodes have significantly higher levels of shame, compared with those patients who only had a single episode (Andrews and Hunter, 1997). In children, it was shown that increased levels of shame responses predicted the severity of major depression in a population of preschool children as young as 3 years old (Luby et al., 2009). This finding is particularly important as it could suggest that shame-proneness in children may also be a vulnerability factor for MDD in later life. Finally, shame reactions and self-blame in the face of stressful life events, such as cancer diagnosis, were associated with MDD diagnosis following the onset of cancer (Hill et al., 2011).

A recent meta-analysis, investigated the relationship between guilt, shame, and depressive symptoms in patients with MDD, as well as individuals self reporting depressive symptoms (Kim et al., 2011). Kim and colleagues showed that there is a stronger relationship between shame and depressive symptoms relative to the association between a generic form of guilt and depressive symptoms. However, they suggested that the relationship between shame and depressive symptoms was not statistically stronger than the relationship between two maladaptive forms of guilt (i.e., omnipotent responsibility guilt and inappropriate/out of context guilt) and depressive symptoms.

### Relationship between guilt and shame

The self-blaming moral emotions of guilt and shame are closely linked. Previous studies have shown significant correlations between context-dependent moral emotions such as guilt and shame (Tangney et al., 1992a), however these constructs can be theoretically distinguished. It is suggested that this distinction mainly relies on participants’ ability to accurately define these moral emotions separately when they experience them within a social context (Tangney et al., 1996). However, patients with MDD may be impaired in their ability to make this distinction. A recent behavioral study provided participants with handheld devices and probed their feelings in the course of daily activities, using items on the Positive and Negative Affects Scale (PANAS), to assess emotions including guilt and shame (Demiralp et al., 2012). The authors showed that patients with MDD were less able to differentiate between negatively valenced emotions; including both guilt and shame. We consider these findings to be particularly important as they suggest that symptomatic criteria for guilt established on the basis of consistent patient accounts in clinical interviews may be insensitive to discriminate guilt from shame. It is possible that the current diagnostic criteria make an overgeneral assumption that pathological self-blame in MDD loads onto guilt, whilst underevaluating the role of shame.

The difficulties in classification of guilt and shame into different categories of moral emotions may partially arise from the limitations of methodologies being used for their assessment (as is often done by asking participants how they would feel in a given hypothetical scenario). Attempts to differentiate between these moral emotions aimed at a distinction based on: (a) types of eliciting events, (b) social audience, and (c) the extent to which a person attributes the failure, within the context of the eliciting event, to the self, or the behavior (Tangney, 1996). Guilt is conceptualized as a more private experience which is reactive to norm violation, leading to behavioral self-blame. On the other hand, shame is referred to as a more public experience which emerges in a wider range of situations which may lead to characterological self-blame (Tangney, 1996). An association of guilt with behavioral, and shame with characterological self-blame is consistent with JanoffBulman’s suggestions of differences between characterological and behavioral self-blame patterns in people with symptomatic subclinical depression (Janoffbulman, 1979). Characterological self-blame is regarded as more depressogenic, as it provides limited space for positive change, whereas self-blame emerging from behavioral violations of one’s moral conduct may subside once that behavior is modified. Two theoretical models supported the characterological versus behavioral self-blame differentiation argument. The revised learned-helplessness model posits that people who suffer from MDD often make internal, stable, and global attributions for negative events (Abramson et al., 1978). In an alternative model, Higgins (1987) suggested a differentiation between guilt and shame based on different patterns of self-discrepancies. Higgins argued that an inability to comply with significant others’ moral standards results in shame, whereas an inability to comply with one’s own moral standards results in guilt (Higgins, 1987).

On the other hand, recent work by O’Connor and colleagues provides evidence against the general association of guilt-proneness with behavioral self-blame by identifying characterological forms of empathy-based guilt (O’Connor et al., 1997, 1999, 2002). Using the Interpersonal Guilt Questionnaire (IGQ-67, O’Connor et al., 1997), which captures characterological forms of empathy-based guilt, they showed elevated scores in symptomatic MDD (O’Connor et al., 2002). Although, empathy-based guilt may be maladaptive for an individual with regard to group competition, it is argued that altruistic individuals with high empathy-based guilt may have provided survival advantages in the competition between groups in our evolutionary history (O’Connor et al., 2000, 2012; Wilson and Wilson, 2007). Empathy-based guilt as measured on the IGQ-67 were associated with depressive symptoms and this association remained even when controlling for levels of shame as measured on Tangney et al.’s scale (O’Connor et al., 1999).

### Functional neuroanatomy of guilt and shame

In this section we will consider social cognitive neuroscience studies investigating the functional neuroanatomy of guilt and shame, and discuss whether these emotions can be distinguished based on their functional neuroanatomy.

Shin et al. (2000) used an autobiographical episodic memory paradigm with positron emission tomography (PET) in order to measure regional cerebral blood flow (rCBF) during guilt-related imagery. In this study, participants rated stimuli in the guilt condition as evoking shame to an extent which was not significantly different than post-scanning guilt ratings, pointing to difficulties teasing apart guilt and shame experimentally (Shin et al., 2000). The authors showed that guilt relative to a neutral condition led to rCBF increase in dorsal anterior cingulate gyrus (BA32) and anterior insula; whilst leading to decreased rCBF in posterior insula. A functional magnetic resonance imaging (fMRI) study conducted in healthy subjects showed that guilt-specific stimuli were associated with significantly increased activation in left posterior superior temporal sulcus (STS) and medial frontopolar cortex (Takahashi et al., 2004). Furthermore, Takahashi and colleagues explored the differences in neural response between guilt and embarrassment (an affective state closely related to shame). They found that embarrassment activated the right anterior temporal lobe and hippocampus bilaterally, compared with guilt. More recent studies concerning the neural response to shame-specific stimuli have been somewhat inconclusive. Wagner et al. (2011) showed that compared with guilt, shame-specific stimuli were not associated with selective activations. In another study, it was shown that in post-scanning ratings, embarrassment, and shame were the second and third most highly descriptive emotions in defining stimuli designed to evoke guilt, as previously suggested by the findings of Shin et al. (2000) and Morey et al. (2012). Firstly, these authors showed that people felt significantly more guilt when their actions affected others rather than themselves. Secondly, they suggested that activation in ventrolateral regions of the prefrontal cortex correlated significantly with post-scanning guilt ratings for actions affecting other individuals. However, they did not provide co-variation analyzes with shame ratings and consequently did not discuss the extent to which this activation may be due to shame or embarrassment. Basile et al. (2010) investigated neurobiological substrates of two other subtypes of guilt: deontological guilt (emerging from violations of one’s own moral conduct) and altruistic (i.e., survivor) guilt. Pairing different guilt scripts with Ekman’s emotional faces in an event-related fMRI design, they showed that deontological guilt activated dorsal and ventral regions of the anterior cingulate cortex (BA 32/24), whereas altruistic/survivor guilt activated frontopolar regions (BA10 and BA9) (Basile et al., 2010). Similar results were also obtained when using script paradigms where significant frontopolar cortex activations were observed for guilt and STS activations for embarrassment in healthy subjects undergoing functional brain imaging (Moll et al., 2007b). Koenigs and Tranel (2007) showed that carers of patients with ventromedial prefrontal cortex (vmPFC) lesions, including the frontopolar cortex (BA10), observed less guilt; providing support for the engagement of frontopolar cortex while people experience guilt. Furthermore, guilt induction by using abstract socio-moral values and showed that activation in the septal and subgenual cingulate region reflected individual differences during the experience of guilt but not indignation whilst controlling for valence, the influence of other emotions such as embarrassment and the psycholinguistic properties of the stimuli (Zahn et al., 2009b). Zahn and colleagues also found the frontopolar cortex to be selectively activated for guilt. In another study, activation in the subgenual cingulate region for guilt relative to a neutral condition correlated significantly with off-line individual empathic concern ratings; an important component of empathic moral sentiments such as guilt and compassion (Zahn et al., 2009a).

Taken together, guilt was most reliably associated with frontopolar activations (i.e., BA10). This result was obtained whilst using various control conditions [other-critical emotions such as indignation (Moll et al., 2007a; Zahn et al., 2009c); anger toward self (Kedia et al., 2008); embarrassment (Takahashi et al., 2004); regret with no consequences for others (Morey et al., 2012); as well as sadness (Basile et al., 2011)]. The subgenual cingulate cortex (sgACC) (including the posteriorly adjacent septal area in some studies) was selectively activated for guilt compared with indignation toward others, when modeling individual variability in empathic concern (Zahn et al., 2009a), or guilt-proneness (Zahn et al., 2009c; Green et al., 2012). Subgenual cingulate activations for guilt were also reproduced by independent groups using quite different ways of inducing guilt (Basile et al., 2011; Morey et al., 2012).

The findings of a study experimentally probing moral sentiments in patients with focal neurodegeneration of anterior brain regions (frontotemporal dementia; FTD) confirmed the fMRI evidence for involvement of the frontopolar and septal region in prosocial sentiments (Moll et al., 2011). Resting-state hypometabolism, as a measure of neuronal dysfunction in the frontopolar cortex and the septal region, was associated with impairments of processing prosocial sentiments whilst controlling for experimentally probed disgust and anger. Whilst frontopolar cortex dysfunction was associated with loss of guilt, pity, and embarrassment; septal dysfunction was specifically associated with loss of empathic moral sentiments (guilt and pity but not embarrassment).

Other research groups investigated the impact of perceived audience on the way people processed moral and social transgressions (Finger et al., 2006). In this study, participants rated guilt to be most relevant to moral transgressions without an audience, whereas highest shame ratings were given to social transgressions with an audience. This differentiation is partially in line with Tangney’s assumption that shame results from moral or social transgressions in the presence of a social audience (Tangney et al., 2007). Comparing their functional neuroanatomy, the authors showed that activation in ventrolateral prefrontal cortex is overlapping for both moral and social transgressions in the presence of an audience (Finger et al., 2006). Another study investigated brain activation changes for social gestures regarded as aversive (fascist salute) relative to a generic greeting (Knutson et al., 2008). The participants were presented with 2 s movies of an adult male performing gestures, as they underwent a brain scan. Separately, and out of the scanner, participants were asked to complete different scales measuring various psychological traits. Here, the authors showed that post-scanning shame ratings correlated significantly negatively with the activation in bilateral inferior parietal lobe for the fascist salute versus the greeting wave.

The first neuroimaging study of guilt in patients with MDD used a psychophysiological interaction (PPI) analysis and reported selective decoupling between subgenual anterior cingulate cortex (sgACC) and right superior anterior temporal lobe for guilt compared with indignation in people with MDD, fully remitted from symptoms (Green et al., 2012). At the time of writing, there are no published studies reporting regions which showed differences for shame responses compared with any other control emotions. More importantly, despite its relevance, there are no published studies addressing the neural response to shame in MDD.

The regions which are critically involved in guilt and shame have an intriguing overlap with regions implicated in depression. Specifically, functional abnormalities in sgACC warrant specific attention. As mentioned previously, the sgACC shows selective activation for guilt. A recent review showed that after partial volume correction for the reduction in gray matter volume, there is abnormal metabolism in sgACC in a patient group relative to controls (Drevets and Savitz, 2008). Various other clinical studies suggested that MDD is associated with structural and functional abnormalities in sgACC (Drevets et al., 1997, 1998; Botteron et al., 2002; Skaf et al., 2002; Greicius et al., 2007; Lehmbeck et al., 2008). Activity in sgACC is also shown to predict treatment outcome in major depression. For example, pre-treatment hypometabolism for negative words in sgACC was associated with successful outcome of cognitive behavioral therapy (Siegle et al., 2006), whereas hypermetabolism predicted better treatment response to selective serotonin reuptake inhibitors (SSRIs) (Mayberg et al., 1997; Keedwell et al., 2010). Subgenual cingulate stimulation was further shown to lead to a remission of major depression (Mayberg et al., 2005).

### Section summary

In this section, we reviewed the literature on moral emotions and MDD. Studies consistently showed that both guilt and shame scores are elevated in patients with MDD. Neuroimaging literature provides insights for understanding overlapping mechanisms between a specific symptom of a disorder (e.g., guilt) and overall pathophysiology. Studies investigating the functional neuroanatomy of guilt and/or shame in patients with MDD are very limited. Current findings suggest that overgeneralization of guilt in MDD is associated with functional disconnection of the anterior temporal cortex and the sgACC, frontopolar, hippocampal, and hypothalamic regions showing guilt-selective disconnection. Previous behavioral studies suggest that there is a need to investigate the functional neuroanatomy of shame in patients with MDD. We provide a summary table of the cited literature for this section (Table 1).

**Table 1:** **The list of the key: (A) literature which investigated guilt and shame in MDD and (B) neuroimaging literature which investigated guilt and shame**.

Reference	Sample	Method	Moral emotion	Scale	Main conclusion
**(A)**					
Demiralp et al. (2012)	MDD	Behavioral	Guilt/shame	PANAS	Inability to differentiate between negative emotions in MDD
Bryan et al. (2013)	MDD	Behavioral	Guilt/shame	Harder personal feelings questionnaire	Shame correlates significantly with depression severity
Bi et al. (2012)	MDD	Behavioral	Guilt	SCID	Excessive guilt in suicide attempters
Kim et al. (2011)	MDD	Meta-analysis	Guilt/shame	–	Significant relationship between shame and depressive symptoms
Marx et al. (2010)	MDD	Behavioral	Guilt	Laufer-parsons inventory	Combat-related guilt mediates MDD diagnosis
Luby et al. (2009)	MDD	Behavioral	Guilt/shame	Story stem task	Shame correlates significantly with depression severity
O’Connor et al. (2002)	MDD	Behavioral	Guilt	IPGQ-67	Survivor and omnipotent responsibility guilt correlates with self-reported severity of symptoms
Berrios et al. (1992)	MDD	Behavioral	Guilt	Novel guilt scale	Guilt scores correlate with self-reported symptoms
**(B)**					
Morey et al. (2012)	Healthy subjects	fMRI	Guilt	Novel guilt scale	Guilt activates dmPFC and vlPFC
Green et al. (2012)	Remitted MDD	fMRI	Guilt/shame	Value related moral sentiments task	Decoupling between sgACC and aTL for guilt in MDD
Wagner et al. (2011)	Healthy subjects	fMRI	Guilt	Trait guilt questionnaire	Guilt activates right OFC
Moll et al. (2011)	Patients with FTD	PET	Guilt	Moral sentiments task	Hypoactivations in the frontopolar cortex and the septal region are associated with impairments in processing prosocial sentiments, including guilt and pity
Basile et al. (2011)	Healthy subjects	fMRI	Guilt	Deontological versus altruistic guilt	Deontological guilt activates dorsal and ventral anterior cingulate, whereas altruistic guilt activates frontopolar cortex (BA 9/10)
Zahn et al. (2009a,b)	Healthy subjects	fMRI	Guilt	Moral sentiments task	Activity in the sgACC in guilt relative to neutral condition, correlates significantly with offline empathic concern ratings
Zahn et al. (2009a,b)	Healthy subjects	fMRI	Guilt	Value related moral sentiments task	Guilt in negative self agency conditions activates septal/sgACC and regions of vmPFC
Moll et al. (2007a,b)	Healthy subjects	fMRI	Guilt	Moral sentiments task	Guilt relative to neutral condition activated frontopolar cortex and STS
Berthoz et al. (2006)	Healthy subjects	fMRI	Guilt	Intentional and accidental moral violations	Guilt activates amygdala bilaterally
Takahashi et al. (2004)	Healthy subjects	fMRI	Guilt	Guilt-embarrassment scale	Guilt activates mPFC and posterior STS
Shin et al. (2000)	Healthy subjects	PET	Guilt	Autobiographical guilt	Increased regional cerebral blood flow in anterior cingulate and anterior insula, and decreased rCBF in posterior insula during guilt versus neutral condition

Studies were listed chronologically (the most recent first), so that advancements and limitations in this field of research can be observed.

## Hypotheses Regarding the Impact of Self-Blaming Emotions on Social-Economical Decision Making

In the previous section, we showed that it was difficult to dissociate the roles of guilt and shame in MDD on the basis of the current behavioral or functional neuroimaging evidence. In social psychology, both guilt and shame are conceptualized to be within the same category of moral emotions which help reduce socially undesirable behavior (Tangney, 1996). However, in a later work they argued that shame contributes less toward motivating moral behavior because its adaptive functions are limited relative to those of guilt (Tangney et al., 2007). They suggested that the main difference between guilt and shame lies in their respective motivations for subsequent action (Tangney et al., 1996). Guilt is associated with an urge for reparative action (Tangney et al., 2007). On the other hand, the most common response to shame is to increase social distance and escape from the shame-eliciting environment. Therefore, it is suggested that shame works counter to reparative altruistic tendencies by means of increasing social distance between individuals (Tangney et al., 2007). The assumptions regarding the reparative nature of guilt find support from clinical research. Considering the significance of both survivor and omnipotent responsibility guilt in MDD, O’Connor and colleagues argue that depression may be conceptualized as a disorder in which the moral system is in “overdrive,” leading patients to pathological forms of altruism and self-sacrifice (O’Connor et al., 2007, 2012). Altruistic behavior has been defined as a form of cooperative behavior conducted at a particular cost to the actor toward an indiscriminate receiver in the absence of short or long term expectancy of reciprocation (West et al., 2007). In conjunction with moral emotions, altruistic behavior can be in the form of direct cooperation or implicated punishment as a result of norm violation. The unique interaction between cooperation and costly punishment is defined as “strong reciprocity,” a concept which incorporates costly cooperation and costly punishment (Fehr and Fischbacher, 2003).

Understanding the relationship between MDD, guilt, shame, and altruism may be more complicated than previously proposed. Both Tangney and O’Connor’s hypotheses have certain limitations. Tangney’s hypotheses are derived mainly from undergraduate student populations, which limits their validity for patients with MDD, whereas O’Connor’s hypotheses may underestimate the role of shame in interpersonal decision-making in MDD. Both of these assumptions rely on self-reported hypothetical behavior and experience, but not actual decision-making. Although one may argue for a direct relationship between choice preferences and actual behavior, the results of choice behavior paradigms are usually confounded by factors such as social desirability. Therefore, computerized behavioral paradigms may have more ecological validity in terms of modeling how people interact with their social environment. Here, we propose a neuro-behavioral economical approach to investigate the extent to which guilt and shame influence social and financial decisions in patients with MDD.

## A Brief Introduction to Neuroeconomical Paradigms

Neuroeconomical paradigms make use of quantifiable resource tangibles such as time, money, or amount of water to survive, so that they activate daily life decision-making mechanisms. These experiments are referred to as “games.” Neuroeconomical game is defined as; “a decision problem with structure so that one’s payoffs can depend on one’s own choices and some other input” (Kishida et al., 2010). These features are suggested as making neuroeconomical paradigms an ecologically valid, interdisciplinary, and empirically testable framework for understanding social impairments associated with neuropsychiatric disorders (Brune, 2002). Recently, it was suggested that average responses of individuals without any psychopathology in various neuroeconomical decision-making paradigms may be used to design realistic social partners for computerized tasks in order to define benchmarks of normative behavior (King-Casas and Chiu, 2012). In the next step, behavioral and neurobiological deviations from the benchmark social norm may be used as quantitative biomarkers to support clinical diagnoses. A neuroeconomical approach may also facilitate translational approaches, as neuroeconomic paradigms can capture elements of social hierarchical organization, that is readily observable in animals, but traditionally harder to evaluate in humans. Indeed authors have used neuroeconomical paradigms in order to explain optimal foraging behavior in the wild (Dubois and Giraldeau, 2003). Although the amount of current scientific input from clinical populations is extremely limited, these recent suggestions are important in highlighting the potential of neuroeconomical paradigms as a cornerstone for the future of psychiatric research.

In the next section, we will consider studies which have investigated the impact of guilt, shame, and depression on social-economical decision-making, reviewing evidence from behavioral, neuroimaging, and neuromodulatory studies in clinical, subclinical populations, and healthy subjects. Our main emphasis will be on neuroeconomical paradigms investigating various dimensions of altruistic behaviors as an important component of social and moral decision-making.

## The Impact of Self-Blaming Moral Emotions on Social-Economical Decision Making

Previous studies have suggested that exogenous manipulations of emotional context influence judgments about acceptability of moral violations (Valdesolo and DeSteno, 2006). Later studies showed that emotions with similar valence, but different psychological properties, have different impacts on the way people make moral judgments (Strohminger et al., 2011). Here, we will discuss the diverging effects of self-blaming moral emotions on social-economical decision making. In the later subsections, we will consider evidence regarding the impact of depression and neurotransmitter modulations.

### The role of guilt

As discussed, previous literature suggests that guilt promotes altruistic behavior. In this section we will consider neuroeconomical evidence regarding the impact of guilt on various dimensions of altruistic behavior. A recent study has shown that individuals with higher levels of guilt-proneness (a trait associated with feeling negative emotions for personal moral violations) consistently made more ethical choices across different domains (Cohen et al., 2012). These individuals were less likely to engage in unethical business decisions such as violating a legal loophole, less likely to lie for financial gain, or in business negotiations, and less likely to engage in counter-productive behaviors at the workplace.

**Figure 1:**
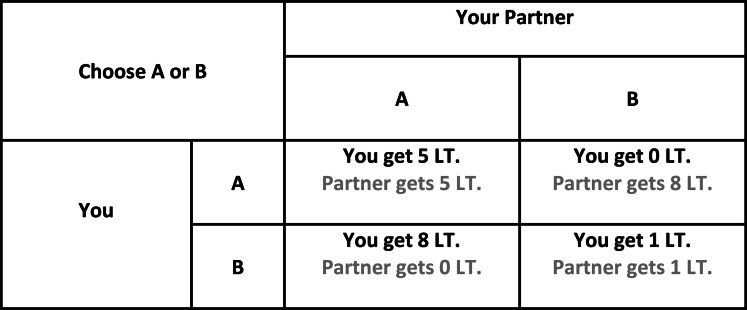
**Schematic diagram of Prisoner’s Dilemma**. In iterated games, players are forced to choose between cooperation and defection in each round blindly to the other player’s choice. Adapted from Mokros et al. (2008).

The so-called “Prisoner’s Dilemma” (PD) is the most frequently used neuroeconomical paradigm to investigate interpersonal cooperation in uncertain environments. Most commonly in PD, participants interact with a single partner over a one-shot or an iterated decision between cooperation and defection, in which the payoffs for any combinations of these decisions are determined on a pre-defined matrix (see Figure 1). The main uncertainty in the environment is derived from the lack of information regarding the intentions of the partner. It was shown that communication prior to the experiment in PD increases the frequency of cooperation (Ostrom, 2006). One study investigated the emotional reactions of responders in the PD where communication was allowed before the experiment. It was shown that individuals felt significantly more guilt upon violating a previous agreement to cooperate, especially when their partners cooperated (Miettinen and Suetens, 2008). Another study investigated the impact of moral emotions on interpersonal cooperation in PD. Using computer simulations, the authors showed that in mixed populations (with equal numbers of altruists and defectors) moral emotions are important for a social group’s survival (Bazzan et al., 2002). However, it is important to emphasize that these computational models consider general principles (i.e., capacity to acquire moral emotions) as opposed to specific involvement of guilt. Another laboratory-based study showed that manipulating guilt in an experimental design increased corporation in PD (Ketelaar and Au, 2003). The effect of guilt induction was especially significant for uncooperative individuals when they interacted with a cooperative strategy (*tit for tat*).

Polman and Ruttan (2012) have recently asked whether guilt had any impact on the way people make moral judgments when evaluating the behavior of others versus themselves. They defined moral hypocrisy as people’s tendency to judge others more severely than they would judge themselves (Polman and Ruttan, 2012). Intriguingly, following guilt-induction, participants rated their own moral violations as less acceptable, whereas they acted more fairly when judging the moral violations of other individuals. This study suggests that guilt may have a fine-tuning role in adjusting perceptions regarding fairness norms. In neuroeconomics, the Dictator Game (DG) is the most frequently used paradigm to evaluate how strongly people endorse the fairness norm. This paradigm is based on an individual’s decision in a single shot interaction with another person in which the dictator (the active participant) proposes to split an amount of money received from the researcher (e.g., for £10; any split above £7 versus £3 is considered as a fair offer) (Hoffman et al., 1996). In the DG, the responder is absolutely passive and cannot decline the proposed amount, whereas any proposal can be declined in the Ultimatum Game (UG). This difference makes the DG a behavioral method for monetary quantification of the fairness norm for the proposing dictator by the amount of money being offered, whereas the Ultimatum bargaining models an interaction between two individuals negotiating for usually an imbalanced equilibrium point of fairness, proverbially trying to establish a point that is “fair enough.” In the UG, the proposer receives a sum of money from the bank (often the researcher) and is asked to propose usually an uneven split. If the responder accepts the split, the money is distributed as proposed. If the responder rejects the split both sides get nothing. The rational actor model (Gintis, 2007) suggests that individuals should be inclined to accept unfair offers in the UG as any financial gain is better than zero gain (Loewenstein et al., 2008). However, empirical evidence suggests that responders only accept around 50% of the unfair offers (defined as proposals which offer less than 30% of the total sum) (Harle et al., 2010). In the altruism literature, the amount of money responders sacrifice in order to punish unfair proposers in the UG is regarded as “altruistic punishment.” Anthropological research using predictive computer simulations showed that the frequency of cooperation can only be maintained in larger groups of individuals when there is costly, altruistic punishment (Boyd et al., 2003). In these simulated environments altruistic punishers, who share a similar payoff with the cooperator but also incur the costs of punishment, were the stabilizing agents in sustaining the level of cooperation (Boyd et al., 2003). Therefore, altruistic punishment is an important domain of altruism, which requires special attention.

Early studies reported that the influence of negative emotions on rejection of unfair offers is significant even after a cooling off period lasting 1 h (Bosman et al., 2001). Experimentally induced guilt by recalling guilt-evoking autobiographical memories leads to increased offers in the UG in a population of healthy volunteers (Ketelaar and Au, 2003). Furthermore, the effects of guilt emerging from previous unfair offers proposed to an assigned partner in the UG extend over time and yield similar results of increased offers in subsequent encounters with the same partner (Ketelaar and Au, 2003). The latter findings suggest that participants display an extended reparative tendency to counter the effects of the initial guilt-eliciting interaction. Similar results were reported by another study which showed a significant correlation between anticipated guilt and the amount of money the proposers offered in the UG (Nelissen et al., 2011). These authors have also shown that Ultimatum offers were significantly higher following a guilt-induction procedure by recalling autobiographical events, confirming previous findings.

We consider charitable donation behavior to be another important domain of altruism. Charitable donation behavior explores individuals’ interactions with the social environment by measuring their responsiveness to various societal causes weighed against their selfish monetary interest. It differentiates from the previously reviewed tasks (PD, DG, and UG) in two respects. Firstly, the financial impact of charitable donation behavior is far reaching, relative to the other paradigms which are based on the interaction between two individuals. Secondly, it measures individuals’ responsiveness to concrete societal causes rather than a universally accepted abstract norm such as “fairness” on which the altruistic punishment in the UG is based. In the case of charitable donations, survivor guilt may motivate individuals to restore a sense of fairness with respect to a specific societal issue, by making donations to those who are in need (e.g., it is not fair that children in Africa are suffering from malnutrition). A recent study showed that guilt induction, irrespective of health concerns, significantly increased the amount of money people were willing to donate to cancer research (Polman and Ruttan, 2012). Secondly, the people who underwent guilt-induction showed similar preferences for how much other individuals should be donating to the same cause. There may be parallels between charitable donation behavior observed in the laboratory and real-life donation behavior; especially in the case of organ donations. It was recently shown that anticipated guilt is one of the key factors influencing people’s decisions to register as organ donors (Wang, 2011). However, another study screening kidney donors, showed that one in five individuals reported clinically significant levels of depression following their donation (Wiedebusch et al., 2009). In post-operative cases, survivor guilt may be an important construct for further investigation, regardless of whether potential research participants are donors or receivers. It has been proposed that survivor guilt operates with similar principles to that of a mathematical construct called “the zero-sum game” which defines limited availability of resources (Blacher, 2000). Under certain conditions, post-operative patients may report clinically significant depression when they perceive their current state of well-being (i.e., surviving a traumatic event) as achieved at the expense of another person’s misery (i.e., only limited number of people can survive) (Blacher, 2000). In these situations, the individuals receiving donations are prone to experiencing survivor guilt, whereas for generic charitable donations behavior survivor guilt may motivate individuals to provide financial help to those who are in need. On the other hand, it is important to highlight that the interpersonal bargaining games that we reviewed in this section (such as UG and DG), adhere to the rules of zero-sum games and therefore, these paradigms may also be effective for probing survivor guilt when proposers decide to keep the bigger proportion of the stake. Considering other studies which showed that making charitable donations is important for psychological well-being (Anik et al., 2009), it is hard to come up with a uniform model for donation behavior which is solely driven by either negative or positive emotions and explain donation behavior across different modalities. Whilst conceptually discussing the impact of personal motivations on altruistic behavior, helping behavior resulting from a need to feel “warm glow” or ward off negative emotions can was framed as impurely altruistic (Andreoni, 1990).

### The role of shame

The impact of shame on social-economical decision making has been widely neglected as a research question. One study investigated the threat of shame in anonymous public goods game and showed that it led to higher levels of contributions to the common pool (Jacquet et al., 2011). A public goods game is played in a multiplayer environment in which each player contributes a proportion of their endowment to a common pool. The amount collected in the common pool is escalated by a pre-defined factor and then evenly distributed back to the players. In these experiments, Jacquet et al. (2011) manipulated the threat of shame by revealing the identity of two of the least generous/cooperative individuals. In a more recent study, the same research group argued that the evolutionary/moral function of a threat of shame is comparable to costly altruistic punishment and it has a fundamental role in sustaining cooperation in society (Jacquet et al., 2012). Following Tangney’s hypothesis regarding the negative influence of acute shame on altruistic behavior, de Hooge et al. (2007) asked whether guilt and shame have opposing effects on interpersonal cooperation. They used an interpersonal donations paradigm in which the value of the endowment was escalated for the donatee (de Hooge et al., 2007). They showed that guilt promoted cooperation only in selfish individuals, whereas they did not provide any support for shame to be reducing altruism, contrary to Tangney’s hypothesis. In a later work, these authors aimed to differentiate the prosocial influence of shame by investigating the nature of the shame-eliciting event and its relevance to the decision-making task (de Hooge et al., 2008). They raised a general question about mood induction paradigms and suggested that emotions triggered by an external cause (i.e., exogenous) should have limited influence on decision-making. These exogenous manipulations include experimental conditions when participants are manipulated to feel an emotion that would not normally arise from the decision-making context. Using the same interpersonal monetary donations game, they show that only endogenous shame lead to significantly higher levels of contributions only in selfish individuals, regardless of whether it was imagined, recalled, or currently experienced.

Earlier studies manipulated the social distance between interacting individuals. Increasing social distance as a result of escaping from a shame-eliciting situation has been purported as one of the behavioral manifestations of incidental shame (Tangney et al., 2007). It has been shown that an experimentally increased social distance by increasing the level of anonymity between players in the DG resulted in lower proposals in a population of healthy volunteers (Hoffman et al., 1996). This study suggests that increasing the social distance leads to compromises in the perception of fairness and actual cooperative behavior. This finding supports one hypothesis proposing that shame serves to reduce altruistic behavior (Tangney et al., 1996) through mechanisms of increasing social distance (Tangney et al., 2007). De Jong et al. (2002) investigated whether the physiological manifestations of shame communicated intentions to sustain cooperation in the PD. They suggested that prosocial individuals who were forced to defect had significantly higher levels of self-reported shame, skin conductance, and face coloration response (De Jong et al., 2002). However, the intensity of these physical manifestations was not sufficient to maintain the social credibility of the defecting individuals in the eyes of the victim of the defection. Another study compared participants’ reactions to unfair proposals offered by either human or computer partners (van’t Wout et al., 2006). Overall, individuals accepted unfair offers at a higher rate when they came from computer partners. Furthermore, rejection rates for the unfair offers correlated significantly with skin conductance activity when these offers came from human partners. Considering the physiological manifestations of shame, this study may suggest that shame could also promote altruistic punishment in healthy subjects.

We have previously mentioned that healthy functioning of moral emotions can only be conceived within an appropriate context. For example, blaming oneself following unfair treatment would be pathological. This issue was later investigated in healthy subjects and it was shown that participants did not display any feelings of shame relative to other outwardly directed negative emotions such as envy, anger, irritation, or contempt following unfair offers in the UG, in line with our perspective (Bosman et al., 2001).

### The impact of depression

The debate surrounding the relationship between altruistic behavior and depression has been mostly theoretical and best to our knowledge, there is no neuroeconomical evidence to support it. The main limitation of the pathological altruism hypothesis is that it does not differentiate between different types of altruism as we have reviewed in the previous sections. Secondly, the majority of the studies supporting this hypothesis are based on self-reported behavioral tendencies as opposed to actual behavioral choice in neuroeconomical paradigms. For example, one retrospective survey suggested that making charitable donations is associated with vulnerability to MDD (Fujiwara, 2009). In this study, the author followed up people who participated in a national survey 10 years ago, and assessed individual tendencies for altruistic behaviors via an e-mail survey. According to the results of the survey, Fujiwara claims that providing emotional and financial support exceeding $10 per month constitutes harmful effects which may be considered as a risk factor for MDD. Although survey studies reach out to larger populations, they may bear significant confounding factors such as social desirability or inaccurate responses. We propose that neuroeconomical paradigms, together with studies investigating self-reported behavioral tendencies, would improve our understanding of social impairments in MDD. Similar opinions have been recently expressed by other authors (Ernst, 2012).

Earlier studies using the PD showed that individuals with self-reported depressive symptoms defected at a significantly higher rate when they were assigned to a high-power role, exploiting the vulnerability set by the individuals in the low power role (Hokanson et al., 1980). In this experiment, the individuals in the low power role were asked to make a decision openly and before the individuals in the high-power role, therefore making them vulnerable to defection. However, another study reported that people with depressive symptoms were more likely to give an aggressive response to betrayal in the PD compared with healthy controls (Haley and Strickland, 1986). This pattern may suggest that individuals with depressive symptoms process moral violations differently when they are the agent or the victim of such violations. Despite higher levels of negative emotions in the face of defection in the PD, one study showed that patients with MDD had significantly higher acceptance rates for unfair offers in the iterated UG, punishing unfair players less than healthy subjects would do (Harle et al., 2010). However, a more recent study showed no significant differences between patients and healthy subjects in terms of acceptance rates, although patients made more frequent fair proposals when they were playing as the proposer (Destoop et al., 2012). Taken together, these findings support the view that following undesirable social outcomes, patients with MDD may feel negative emotions more intensely, yet under certain conditions these emotions may not translate into punishing behavior. There are few previous studies which investigated the impact of affective states on Ultimatum bargaining. Bosman et al. (2001) investigated the impact of emotions on rejecting unfair offers in the one-shot UG in healthy subjects. They showed that sadness was significantly higher in subjects who rejected the unfair proposals compared with those who accepted them. Further, they showed that the intensity of sadness significantly increased the probability of unfair offers being rejected. In their earlier work, Harlé and Sanfey (2007) questioned the impact of acute sadness on decision-making in the UG. They showed that incidental sad mood disturbed mechanisms associated with a rational actor model of decision making, and participants who underwent sad mood induction rejected unfair offers more frequently (Harlé and Sanfey, 2007). We think that findings reported by Harlé and Sanfey are important as they suggest that impairments in social decision-making mechanisms in patients with MDD are different to the impairments caused by external mood induction.

As is evident from our review, social-economical decision-making studies in MDD are very limited, and it is certainly not possible to argue for selective deficits in neuroeconomical decision-making. The other area of psychopathology where these paradigms have been used is psychopathy. Interestingly, a lack of guilt has been hypothesized in psychopathy which is directly opposite to the proposed exaggerated manifestation of guilt in MDD. The findings which we will briefly review below may suggest that neuroeconomical paradigms are sensitive to detecting these opposing aspects of psychopathology. For example, it was shown that individuals with high psychopathic tendencies accepted unfair offers in the UG at a significantly higher rate than healthy subjects (Osumi and Ohira, 2010). The authors proposed that the affective impairment in individuals with high psychopathic tendencies, as measured by skin conductance, is not necessarily a maladaptive one but can be linked to successful adaptations to the social environment (Harpending and Sobus, 1987; Osumi and Ohira, 2010). Another study compared primary and secondary psychopaths with healthy subjects, and showed that only primary psychopaths displayed hypervigilant punishing behavior in the UG (Koenigs et al., 2010). This finding highlights the possibility of using neuroeconomical measures to probe aberrant social decision-making in clinical populations, potentially revealing different profiles within the same clinical diagnostic group. Behavior of inpatient psychopaths has also been associated with a higher payoff defecting strategy in a social variant of the PD in which participants interacted over daily rations of water in a survival situation (Mokros et al., 2008). The authors suggested that the game behavior validly reflects real-life decision making and that the amount of environmental rewards obtained by the inpatients may reduce motivation for recovery.

### Neuromodulatory and neuroimaging studies

It is possible that social impairments associated with MDD have neuronal origins. However, whether these impairments are caused by structural differences in cytoarchitecture existing before the onset of MDD, or caused by the scaring effect of recurrent episodes remains unknown. It was recently shown that in asymptomatic patients with MDD there is hypoactivation to pictures of social interactions (irrespective of valence) in regions of the brain associated with behavioral planning (Elliott et al., 2012). This may suggest that social decision-making deficits reviewed in the previous sections may originate from disruptions in neural networks purported to mediate social perspective taking and planning. In this section we will consider evidence from neuroimaging and neuromodulatory studies in order to identify potential neuronal correlates of moral emotions in depression.

Zak proposed a triangular model in which he considered the influence of oxytocin, serotonin, and dopamine on guiding human social behavior. He suggested that these neurochemicals act selectively on affiliative bonding, mood states, and reward mechanisms respectively (Zak, 2011). In successive UG experiments, it was shown that oxytocin promoted generosity and increased the magnitude of the UG offers significantly, whereas inhibiting oxytocin binding reduced both generosity and the magnitude of the offers, while increasing the threshold for altruistic punishment (Zak, 2011). Another study showed that a genetic polymorphism associated with the oxytocin receptor may have a direct influence on the amount of money people offered in DG interactions (Israel et al., 2009), providing further support for the role of oxytocin in guiding such altruistic decisions. Later studies investigated whether oxytocin enhances different kinds of behaviors associated with altruism. It was shown that intranasal oxytocin infusion promotes hyper-altruism toward in-group-members and territorial/defensive aggression toward out-group members; a behavioral strategy defined as parochial altruism (De Dreu et al., 2010). Impact of oxytocin on in-group hyper-altruism behavior was reproduced whereby individuals having oxytocin infusion significantly increased the amount of money they donated to humanitarian charities providing help to their in-group-members (Barraza et al., 2011). In healthy subjects undergoing brain scan, it was recently shown that oxytocin augmented caudate response to reciprocated cooperation, possibly enhancing the neural response to social rewards (Rilling et al., 2012). Rilling et al. (2012) also showed that oxytocin augments left amygdala activation to altruistic decisions and further enhancing functional connectivity between amygdala and anterior insula. Taken together, these studies provide support for the argument that oxytocin promotes altruistic behavior by enhancing affiliative feelings.

Other studies investigated the impact of acute manipulation of serotonin (which is one of the key neurotransmitters implicated in MDD) on interpersonal cooperation in the PD as well as altruistic punishment in the UG. It was shown that acute tryptophan depletion significantly impaired interpersonal cooperation in healthy subjects. Participants in a PD game showed reduced cooperation with playing partners who they were encountering for the first time, but not during subsequent encounters (Wood et al., 2006). In UG studies, it was shown that lowering serotonin levels by acute tryptophan depletion increased the frequency of altruistic punishment, whereas serotonin loading by acute citalopram increased the frequency of acceptance of unfair offers (Crockett et al., 2008, 2010). Also considering the evidence previously reported by Harlé and Sanfey (2007), the UG studies suggest that profiles for altruistic punishment between patients with MDD, individuals who underwent low mood induction and neurotransmitter manipulation are different from each other.

In their seminal study, Greene et al. (2001) showed that personal (taking active physical role) moral violations activated the frontopolar cortex (BA 10) significantly relative to non-moral conditions. Although this study was very influential in triggering scientific interest in the neural basis of moral reasoning, the dilemmas used in this study and many subsequent others, force individuals to choose between two outcomes involving harming somebody. A typical example is the “runaway train dilemma” where a train is rapidly approaching five people, but pushing a single individual onto the tracks would bring the train to a stop saving the five (Greene et al., 2001). Participants must choose whether or not to push the individual onto the track. Although such dilemmas may produce robust activations by triggering utilitarian evaluation of the value of life, they have limited correspondence to our everyday lives. Therefore, we think that studies using interpersonal cooperation, bargaining, and donation paradigms have more ecological validity in understanding the neural basis of social perception and moral decision-making. In the PD paradigm, reciprocal cooperation is associated with activations in nucleus accumbens, caudate, vmPFC, and ACC, whereas deactivations in ACC and dorsolateral prefrontal cortex (dlPFC) were associated with defection when the partner cooperated; both of these activations correlating significantly with self-reported psychopathy scores (Rilling et al., 2002, 2007). Another study showed that during reciprocal exchange, altruistic individuals activated the frontopolar cortex more when they interacted with human partners compared with computer partners (McCabe et al., 2001). In the UG paradigms, receiving unfair offers activated the dorsal section of the ACC, whereas activity in the right anterior insula correlated significantly negatively with the frequency at which the unfair offers were accepted (Sanfey et al., 2003). One subsequent study, using PET imaging in an interpersonal reciprocal exchange paradigm, showed that healthy subjects activated the right dorsal caudate nucleus when they could effectively punish unfair individuals (de Quervain et al., 2004).

The role of the dorsal ACC in evaluation of players in interpersonal economic exchanges has been a topic of interest as such evaluations are important in guiding decisions whether or not to punish unfair players. During their evaluation of the active participants, observers activated anterior insula and ACC when fair participants received unfair electric shocks, whereas activations related to empathic concern were significantly reduced in male participants when they observed unfair players receiving electric shocks (Singer et al., 2006). The involvement of dorsal ACC and right anterior insula in altruistic punishment was reproduced by a more recent study using the impunity game paradigm, which is a variant of the UG in which the amount the proposer designated for him/herself is immune to the punishment of the responder (Takagishi et al., 2009).

The lesion literature suggests that ventral parts of the prefrontal cortex are involved in neuroeconomic decisions. Koenigs and colleagues showed that vmPFC is an important region guiding altruistic punishment decisions in the UG (Koenigs et al., 2010). Similarly in patients with bilateral vmPFC lesions, it was shown that patients offered significantly less in the DG and demanded significantly more than what they offered in the UG (Krajbich et al., 2009). Considering the lesion evidence about functional neuroanatomy of guilt in the vmPFC, it is possible that a diminished sense of guilt disrupts regulatory mechanisms which influence these interpersonal financial decisions. Experimental studies showed that disrupting brain activation may have influence on altruistic punishment decisions. Using transcranial magnetic stimulation (TMS), which employs an external electromagnetic pulse to disrupt brain activity in cortical regions, Knoch et al. (2006) demonstrated that temporarily disrupting function of right dlPFC diminished altruistic punishment without changing perceptions about the fairness of proposals only when participants interacted with human partners. Another similar methodology is transcranial direct current stimulation, by which brain activity in certain cortical regions is disrupted by delivering a low current through external electrodes. Using this methodology, results obtained by TMS were reproduced, highlighting the role of the right dlPFC in guiding such altruistic decisions (Knoch et al., 2008).

Charitable donation paradigms are also important for neuroeconomical studies. Moll et al. (2006) not only explored the functional neuroanatomy of mechanisms which modulate decisions to make donations, but also those associated with decisions to “punish” charitable organizations when they work counter to participants’ social and moral values. This study managed to incorporate both aspects of human altruistic behavior (costly cooperation and altruistic punishment), also defined as “strong reciprocity” (Fehr and Fischbacher, 2003). They showed that septal and subgenual cingulate regions showed selective activation for decisions to donate relative to a pure monetary reward condition, whereas the activity in ventral striatum was shared for donation and monetary reward. Furthermore, they showed that decisions involving opposition activated lateral orbitofrontal regions and anterior insula bilaterally (Moll et al., 2006). Finally, they showed that activation in the anterior orbitofrontal and frontopolar cortices correlated significantly with the amount of real-life altruistic engagement. Subsequent studies demonstrated that ventral striatum and the septal region showed activation irrespective of whether the donations were voluntary or not (Harbaugh et al., 2007). More recently, a study showed significant ventral striatum activity for social approval when people make donations in the presence of a social audience, providing further information about the role of ventral striatum in social reward processing (Izuma et al., 2010). Izuma and colleagues did not report any specific regions for selfish decisions in this donations paradigm. However, we think that this paradigm may be effective in probing guilt when people make selfish decisions when the social audience is absent; and shame, when the selfish decision is made in the presence of a social audience. Finally, in an interpersonal charitable donations paradigm it was shown that selfish monetary decisions deactivated vmPFC even when these decisions were more equitable based on the evaluation of the magnitude of the financial reward (Zaki and Mitchell, 2011). Considering the previous evidence regarding the role of vmPFC in guilt processing, it is possible to speculate that such selfish decisions required inhibition of guilt. Secondly, the reason why this study did not report any striatal activation may be because helping random individuals who do not give any distress signal may not be sufficient to activate regions selective for socially rewarding decisions, which is required for altruistic behavior.

### Section summary

**Figure 2:**
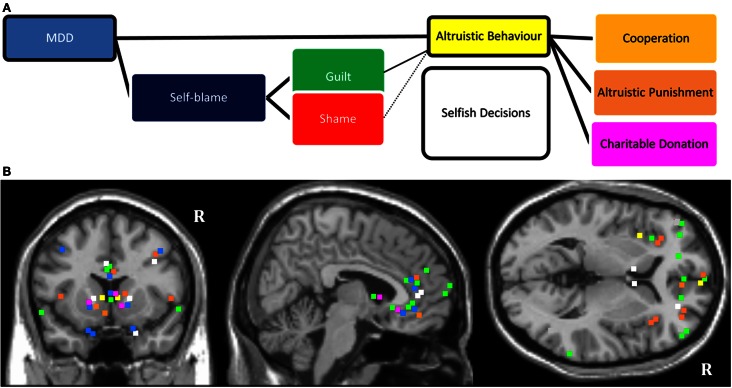
**(A)** Schematic diagram showing influence of MDD on altruistic behavior. The model considers the impact of the mood state along with abnormally elevated self-blaming feelings (guilt and shame). The proposed model also considers different types of altruism, such as cooperation, altruistic punishment, and making donations. **(B)**. Mapping of MNI coordinates of the peak region of activations of the reviewed neuroimaging literature. The studies which were included were not selected based on a systematic review of the literature. The color coding refers to the colors in **(A)** (e.g., green marks specifying activations selective for guilt). “R” denotes right hemisphere. There may be slight distortions when converting 3D images onto 2D T1 structural anatomical template. All mapping remains accurate within structural neuroanatomical label of the regions. Reviewed evidence suggests that frontopolar, ventromedial, right dorsolateral PFC; dorsal and subgenual ACC, striatum and amygdala are important regions of interest (ROIs) for studying affective disturbances and social-economical decision making in MDD.

In this section, we have reviewed literature (summarized in Table 2) investigating the influence of self-blaming moral emotions and depression on decision-making in neuroeconomical paradigms. The studies with guilt-induction consistently show that guilt promotes altruistic behavior, whereas the findings are somewhat ambiguous for studies with shame induction. There are very few studies conducted in patients with MDD. We suggest that MDD diagnosis and accompanying elevation in self-blaming feelings exert an influence on altruistic behavior, as shown schematically in Figure 2A. Functional neuroimaging studies suggest that decisions in neuroeconomical paradigms activate regions of the PFC, insular cortex, and subcortical regions which are purported to mediate reward processing. MDD has been associated with structural and functional abnormalities in these fronto-mesolimbic pathways (see Figure 2B legend).

**Table 2:** **The list of key literature using neuroeconomical paradigms**.

Reference	Sample	Method	Moral emotion	Paradigm	Main conclusion
Polman and Ruttan (2012)	Healthy subjects	Behavioral	Guilt	Donations	Guilt reduces moral hypocrisy
Destoop et al. (2012)	MDD	Behavioral	–	UG	No difference of acceptance frequency between patients and controls
Nelissen et al. (2011)	Healthy subjects	Behavioral	Guilt	UG	Anticipated guilt correlates with the amount of money offered
Jacquet et al. (2011)	Healthy subjects	Behavioral	Shame	Public goods	Threat of shame increased the amount of donations
Zak (2011)	Healthy subjects	Neuro modulation	–	UG	Oxytocin increases offers, inhibiting its binding reduces generosity
Harle et al. (2010)	MDD	Behavioral	–	UG	Patients accept unfair offers at a higher rate than controls
Koenigs et al. (2010)	Psychopaths	Behavioral	–	UG/DG	Higher altruistic punishment but lower dictator offers in primary psychopaths
Izuma et al. (2010)	Healthy subjects	FMRI	–	Donations	Ventral striatum activation for donations in the presence of social audience
Osumi and Ohira (2010)	Psychopaths	Behavioral	–	UG	Psychopathic individuals accept unfair offers at a higher rate
Krajbich et al. (2009)	vmPFC patients	FMRI	–	UG/DG	Patients make lower dictator offers
Miettinen and Suetens (2008)	Healthy subjects	Behavioral	Guilt	PD	Defectors feel guilt when they violate previous cooperative agreements
de Hooge et al. (2008)	Healthy subjects	Behavioral	Shame	Donations	Endogenous shame promotes interpersonal donations
Crockett et al. (2008)	Healthy subjects	Neuro modulation	–	UG	Acute tryptophan depletion increases altruistic punishment
Mokros et al. (2008)	Psychopaths	Behavioral	–	PD	Inpatient psychopaths defect significantly more than controls
de Hooge et al. (2007)	Healthy subjects	Behavioral	Guilt/Shame	Donations	Guilt promotes interpersonal donations, shame does not have any impact
Rilling et al. (2007)	Subclinical	FMRI	–	PD	Deactivation of ACC for decisions to defect
Singer et al. (2006)	Healthy subjects	FMRI	–	PD	Empathic responses in ACC and anterior insula when fair participants are punished with an electric shock
Knoch et al. (2006)	Healthy subjects	Behavioral	–	UG	Disrupting right dlPFC diminishes altruistic punishment
Moll et al. (2006)	Healthy subjects	FMRI	–	Donations	sgACC activation for decisions to make donations
Sanfey et al. (2003)	Healthy subjects	FMRI	–	UG	Right anterior insula activation for altruistic punishment
Ketelaar and Au (2003)	Healthy subjects	Behavioral	Guilt	PD/UG	Guilt increased cooperation and the amount of money offered
Hoffman et al. (1996)	Healthy subjects	Behavioral	–	DG	Increasing social distance reduces dictator offers
Hokanson et al. (1980)	Subclinical	Behavioral	–	PD	Depressed individuals defected more in the high-power role

Studies were listed chronologically (the most recent first), so that advancements and limitations in this field of research can be observed.

## Conclusion and Final Word

In the present opinion paper, we have argued that MDD is associated with elevated proneness to guilt and shame. We highlighted the possible overlap in the functional neuroanatomy of guilt and shame with regions known to be functionally abnormal in MDD, and emphasized a need for more neuroimaging studies to dissociate the functional neuroanatomy of guilt and shame in patients with MDD. Following the suggestions of previous authors, we considered the idea that guilt and shame can be differentiated based on their influence on social-economical decision making. We showed that there is converging literature supporting a positive impact of guilt on altruistic decisions; however literature on the impact of shame is inconclusive. Studies of social-economical decision making to date have focused on healthy populations. Considering coexisting emotional processing abnormalities in MDD, it is more challenging to dissociate the impact of moral emotions on social-economical decision making in clinical populations. In order to address this issue, we have argued in this opinion paper that using functional imaging with neuroeconomical paradigms could be an important direction for future psychiatric research.

## Conflict of Interest Statement

The authors declare that the research was conducted in the absence of any commercial or financial relationships that could be construed as a potential conflict of interest.
